# The Association of IL28B Polymorphism and Graft Survival in Patients with Hepatitis C Undergoing Liver Transplantation

**DOI:** 10.1371/journal.pone.0054854

**Published:** 2013-01-30

**Authors:** Sridhar R. Allam, Bernd Krüger, Anita Mehrotra, Thomas Schiano, Bernd Schröppel, Barbara Murphy

**Affiliations:** 1 Division of Nephrology, Mount Sinai School of Medicine, New York, New York, United States of America; 2 V. Medizinische Klinik, Universitätsmedizin Mannheim, Medizinische Fakultät Mannheim der Universität Heidelberg, Mannheim, Germany; 3 Division of Liver Diseases, Mount Sinai School of Medicine, New York, New York, United States of America; 4 Recanati Miller Transplantation Institute, Mount Sinai School of Medicine, New York, New York, United States of America; The University of Hong Kong, Hong Kong

## Abstract

Hepatitis C virus (HCV) infection is the leading cause of liver transplantation (LT) in Western countries. Polymorphism in the IL28B gene region has a major impact on the natural history and response to antiviral treatment in HCV. We investigated whether IL28B polymorphism was associated with graft survival in patients with or without HCV undergoing LT. 1,060 adult patients (age >18 years) underwent LT between years 2000 and 2008. Patients with previous LT, living donor LT and patients dying or requiring retransplants within 30 days of LT were excluded. DNA samples of 620 (84%) recipients and 377 (51%) donors were available for genotyping of IL28B rs12979860C>T. Donor IL28B genotypes had no significant differences in graft survival irrespective of HCV status. There was no difference in graft outcome in the non-HCV cohort (n = 293) based on recipient IL28B genotype. In the HCV group (n = 327), recipients with CC or CT genotype had better graft survival compared to TT genotype (62% vs. 48%, p = 0.02). HCV recipients with CC or CT genotype had delayed time to clinically relevant HCV recurrence compared to TT (10.4 vs. 6.7 months, p = 0.002). The beneficial effect of the CC/CT genotype on HCV recurrence and graft survival was independent of antiviral treatment. In conclusion, our study demonstrated that in contrast to donor IL28B genotype recipient IL28B was associated with graft survival and clinically relevant HCV recurrence in HCV infected recipients. No effect of IL28B genotype was manifest in non-HCV LT recipients.

## Introduction

Hepatitis C virus (HCV) affects about 4 million people in the United States and nearly 170 million people worldwide [1]. HCV causes 40% of all chronic liver disease in US and HCV-associated cirrhosis is the leading indication for liver transplantation (LT) in the US [2]. Recurrent HCV is the leading cause of graft failure after LT in HCV recipients and treatment with interferon based anti-viral therapy is offered for those with recurrent HCV after LT but bears sub-optimal response rates and is poorly tolerated [3]. It has been reported that an IL28B gene polymorphism was associated with spontaneous clearance and sustained virological response (SVR) to interferon based therapy in general populations affected with HCV [4]–[6]. More recently studies have shown that the IL28B polymorphism was associated with SVR to pegylated-interferon and ribavirin therapy for recurrent HCV after LT [7]–[11].

IL28A, IL28B and IL29, also called type III or lambda interferons (IFN-λ3), are induced by viral infections and are upregulated in hepatocytes and peripheral blood mononuclear cells of individuals with HCV infection [12], [13]. IL28B is encoded by six exons and located on chromosome 19. IL28B interacts with a heterodynamic class II cytokine receptor that consists of IL10Rβ and IL28Rα, resulting in antiviral activity by induction of interferon-stimulated genes through JAK-STAT pathway [12], [14], [15]. IFN-λ3, the product of IL28B gene, was found to regulate Treg and enhance adaptive cellular immunity [16].

Understanding the impact of donor and recipient IL28B variants on post-transplant outcomes might yield novel insights into the mechanism of liver graft failure. Recent studies point to an influence of IL28B variants on response to anti-HCV therapy and HCV recurrence [7]–[11], [17]. Data regarding the effect of IL28B on overall graft survival are scare. Herein, we investigated whether donor and recipient IL28B polymorphism are associated with long-term graft survival in a large cohort of non-HCV and HCV infected LT recipients.

## Materials and Methods

### Study Population

We studied consecutive adult patients (age >18 years) who underwent LT at Mount Sinai Hospital, New York between January 2000 and December 2008. Patients who had retransplants, living donor liver transplants, or patients dying or requiring retransplantation within 30 days of LT (primary non-function, PNF) were excluded from analysis [18]. As standard of care adopted by our center, all patients received IL2 receptor antibody for induction therapy, and were maintained on tacrolimus with or without mycophenolate mofetil and steroid taper (off steroids by 6 months post LT). Samples used for DNA analysis and clinical data on these patients were entered prospectively and analyzed retrospectively. All patients were followed until graft loss, death or the last follow-up visit. Clinical data collected included donor and recipient age, ethnicity and gender, cold ischemia time, presence of hypertension, diabetes and hepatocellular carcinoma (HCC), HCV status, HCV genotype (tested since 2004), HCV viral load (tested since 2004), anti-HCV therapy, Model for End-Stage Liver Disease (MELD) score at the time of LT, cause of liver failure, liver chemistry tests, liver biopsy results, date of death or graft failure. Graft survival data were also obtained from Scientific Registry of Transplant Recipients (SRTR). Liver related graft loss was defined as graft loss occurring secondary to complications of portal hypertension or liver failure directly related to HCV. Non-liver related graft loss was defined as death due to causes unrelated to HCV recurrence or liver disease and with a functioning graft. The study was approved by the Institutional Review Board of Mount Sinai School of Medicine. The IRB waived the need for written, informed consent for this retrospective chart review.

### Definition of HCV Recurrence and Antiviral Therapy

Clinically relevant HCV recurrence was defined as histological evidence of HCV in a liver biopsy that was performed in the setting of abnormal liver chemistry tests. On histology HCV recurrence was defined as≥grade 1 inflammation [19]. Patients who did not undergo liver biopsy were excluded from the analysis of HCV recurrence. Sustained virological response (SVR) was defined as undetectable serum HCV RNA at 24 weeks after the end of treatment with pegylated interferon and ribavirin. Patients were started on therapy if they had≥stage 2 fibrosis on liver biopsy or the cholestatic variant of HCV recurrence. An escalating dose regimen was used for treatment for recurrent HCV consisting of pegylated interferon alpha 2b (90 µg SQ weekly) and ribavirin (400 mg PO daily) and advancing to full dose within 4–8 weeks depending on the tolerability and blood counts. All patients received 48 weeks of therapy regardless of their genotype and despite virological response. Reasons for discontinuation included infection, rejection, severe cardiovascular complications, and severe rash.

### IL28B Genotyping

Recipient DNA samples were obtained from paraffin-embedded explanted recipient liver and donor DNA samples were obtained from paraffin-embedded pre-implantation biopsy of the liver graft. DNA was extracted from paraffin-embedded liver tissue blocks per the manufacturer’s protocol (DNeasy blood and tissue kit, Qiagen Inc, Valencia, CA, USA). IL28B genotyping (rs12979860) was performed using the Taqman Real-Time PCR assay and allelic discrimination kit (Applied Biosystems, Foster City, CA, USA).

### IL28 Gene Expression

For IL28 gene expression, total RNA was extracted according to manufacturer’s protocol (RNeasy FEPE Kit, Qiagen Inc, Valencia, CA, USA) from paraffin-embedded pre-implantation biopsies of donor liver. First-strand cDNA was synthesized using Omniscript RT kit and qRT-PCR was perfomed using QuantiTect SYBR Green PCR kit from the same manufacturer. Primers were designed for IL28 as previously described [20]. The expression levels of IL28 mRNA were normalized to expression of housekeeping gene 18S.

### Statistical Analysis

Results were expressed as means ± standard deviation, unless stated otherwise. T-test was used to analyze continuous variables and chi-square test was used for categorical variables. Hardy-Weinberg equilibrium for genotypes was tested as previously described [21]. Graft survival curves were compared using Kaplan-Meier test and multiple logistic regression method was used for multivariable analysis. The impact of sustained virological response (SVR) or anti-viral therapy on graft outcome was analyzed in a multivariable approach. In the multivariable approach variables with a p<0.2 in the univariable analysis were included. P value of less than 0.05 was considered statistically significant. Statistical analysis was performed using SPSS version 18.0 software package (SPSS Inc., Chicago, IL, USA). Assuming the following for the HCV group: alpha = 0.05, 8 years of accrual time (2000–2008), 5.5 years of follow-up time, and a median survival of 5 years for “controls” (CC/CT genotype), 254 “controls” (CC/CT) and 73 “at-risk” (TT) subjects provides a power of 90% to detect a hazard ratio of 1.75 for survival using conventional Kaplan-Meier survival analysis. Assuming the same for the non-HCV group, 251 “controls” and 42 “at-risk” subjects provides a power of 74% to detect a hazard ratio of 1.75 for survival.

## Results

### Patient Characteristics

**Figure 1:**
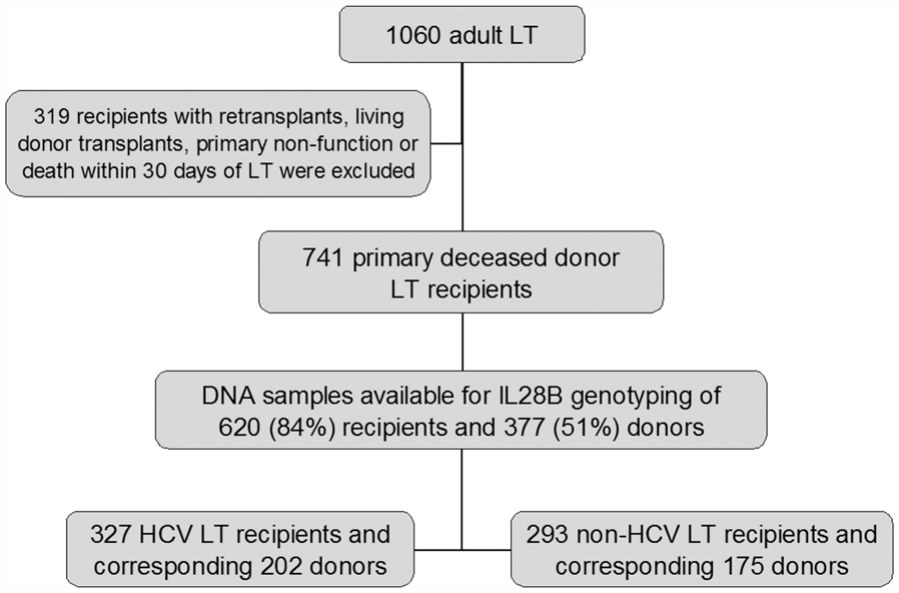
Flow diagram of study participants with available genotyping data. LT = liver transplantation.

1060 adult patients (age >18 years) underwent LT between January 2000 and December 2008. After excluding 319 patients who had retransplants (n = 156), living donor transplants (n = 115), PNF or death within 30 days of LT (n = 48), we included 741 patients who were primary deceased donor LT recipients. Of the 741, DNA samples were available and adequate for IL28B genotyping of 620 (84%) recipients and corresponding 377 (51%) donors. Of 620 recipients, 327 were HCV recipients and 293 were non-HCV recipients. Of 377 donors, 202 were donors for HCV recipients and 175 were donors for non-HCV recipients (Fig. 1). Among recipients without graft loss, follow-up times ranged from 1.5 years to 10.6 years. Mean follow up was 5.5±2.5 years.

Demographic and clinical characteristics, stratified by HCV status and recipient IL28B genotype are shown in table 1. Based on graft survival data, patients were grouped into CC or CT genotype and TT genotype, and demographics were compared between these two groups. Among HCV recipients, there were no significant differences between patients with CC/CT genotype compared to patients with TT genotype. Among non-HCV recipients, proportion of male recipients was significantly higher with genotype CC/CT compared to TT genotype (Table 1).

**Table 1:** Donor and recipient characteristics based on recipient IL28B rs12979860 genotypes.

	HCV			Non-HCV		
IL28B	CC/CT (n = 254)	TT (n = 73)	P value	CC/CT (n = 251)	TT (n = 42)	P value
Recipient age (y)	55.3±8.1	55.4±6.9	0.92	51.5±12.2	53.1±13.3	0.44
Recipient gender, male (%)	75	80	0.44	70	48	0.008
Recipient race, white (%)	69	59	0.12	61	57	0.73
Donor age (y)	48.4±18.6	50.8±18.1	0.36	48.2±20.0	54.8±18.2	0.05
Donor gender, male (%)	58	45	0.06	56	59	0.86
Donor race, white (%)	58	63	0.5	56	62	0.5
Cold ischemia time (hours)	9.2±2.7	9.3±3.7	0.78	9.3±3.0	9.2±3.8	0.83
Hypertension (%)	45	45	0.99	37	31	0.47
Diabetes (%)	29	27	0.82	22	21	0.89
HCC (%)	47	49	0.79	25	33	0.25
HCVgenotype (%) 1a	37	40				
HCVgenotype (%) 1b	24	22				
HCVgenotype (%) 2	5	3	0.67	N/A	N/A	N/A
HCVgenotype (%) 3	4	1				
HCVgenotype (%) 4	2	4				
Mixed genotype	1	0				
Unknown genotype	28	30				
HCV VL (IU/ml) (median, IQR)	321,000(34,150–758,000)	253,000(22,475–950,250)	0.87	N/A	N/A	N/A
VL neg (n, %)	2 (1%)	2 (3%)	0.18			
VL unknown (%)	54%	43%	0.11			
MELD score at the time of LT	28.9±6.3	29.1±5.1	0.79	28.2±7.8	25.4±5.7	0.07

LT = liver transplant; HCC = hepatocellular carcinoma; VL = viral load at the time of LT.

### IL28B Genotype Distribution

Among recipients, genotype frequencies were 37%, 45% and 18% respectively for CC, CT and TT genotypes. Among donors, genotype frequencies were 34%, 45% and 21% respectively for CC, CT and TT genotypes (Table 2). These frequencies were in Hardy-Weinberg equilibrium. CC genotype was significantly less prevalent in HCV compared to non-HCV recipients (31% vs. 47%, p<0.001). Within the HCV group, there was no significant difference in prevalence of CC genotype between recipients and donors (31% vs. 33%, p = 0.36).

**Table 2:** Frequencies of donor and recipient IL28B rs12979860 genotypes in patients with or without HCV.

	Recipients		Donors	
IL28B	HCV	Non-HCV	HCV	Non-HCV
CC	96 (31%)	132 (47%)	66 (33%)	61 (35%)
CT	158 (47%)	119 (39%)	84 (42%)	85 (49%)
TT	73 (22%)	42 (14%)	52 (25%)	29 (16%)

### Recipient IL28B Genotype was Associated with Graft Survival

**Figure 2:**
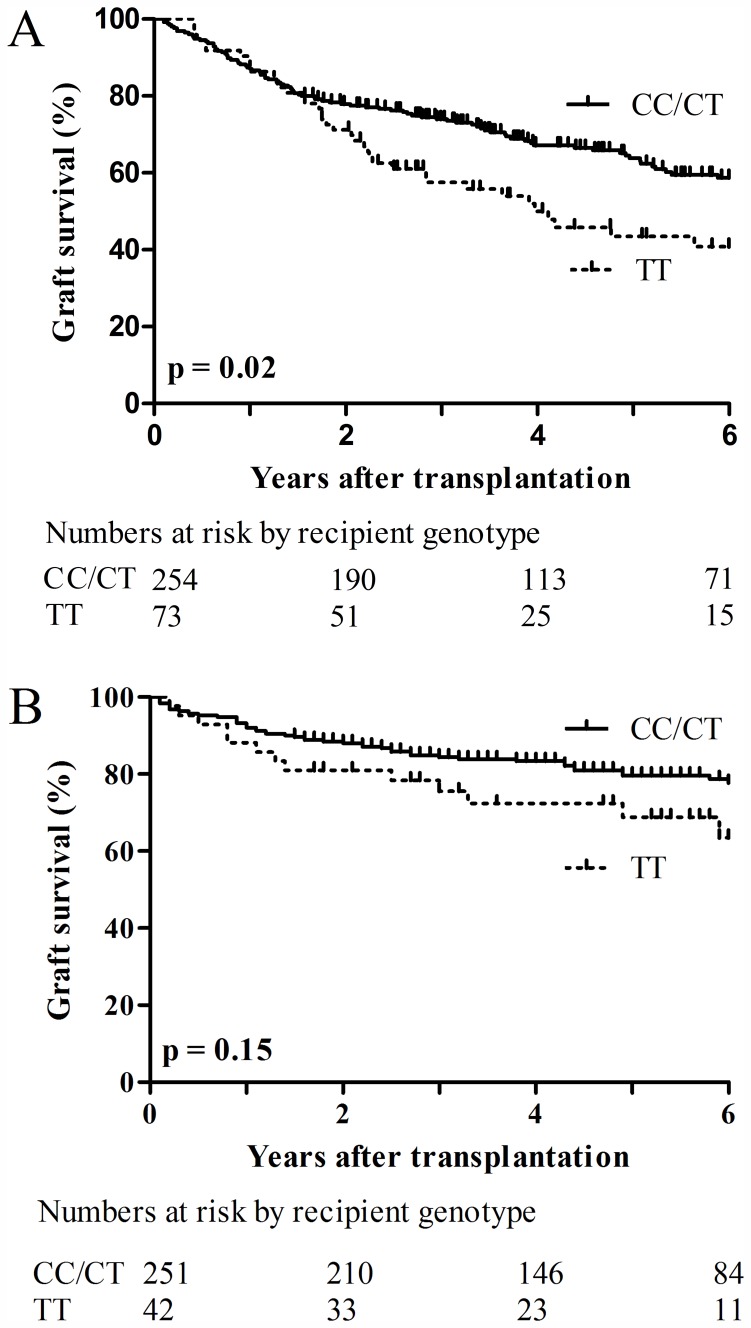
Recipient IL28B genotype and graft survival by HCV status. Kaplan-Meier curves for liver graft survival in HCV infected (A) and non-HCV recipients (B).

Among HCV recipients, overall graft survival rates were 61%, 62% and 48% respectively for patients with CC, CT and TT genotypes. Given similar survival between CC and CT genotypes, survival curves were plotted comparing the group with recipient IL28B CC/CT and the TT. Graft survival was higher in CC/CT compared to TT recipients (62% vs. 48%, p = 0.02, Fig. 2A). Non-liver related graft loss included infection/sepsis (75%), cardiac events (9%), cancer (5%), (CVA (3%), MVA (1.5%), suicide (1.5%), GVHD (1.5%), PTLD (1.5%), PML (1.5%).

**Figure 3:**
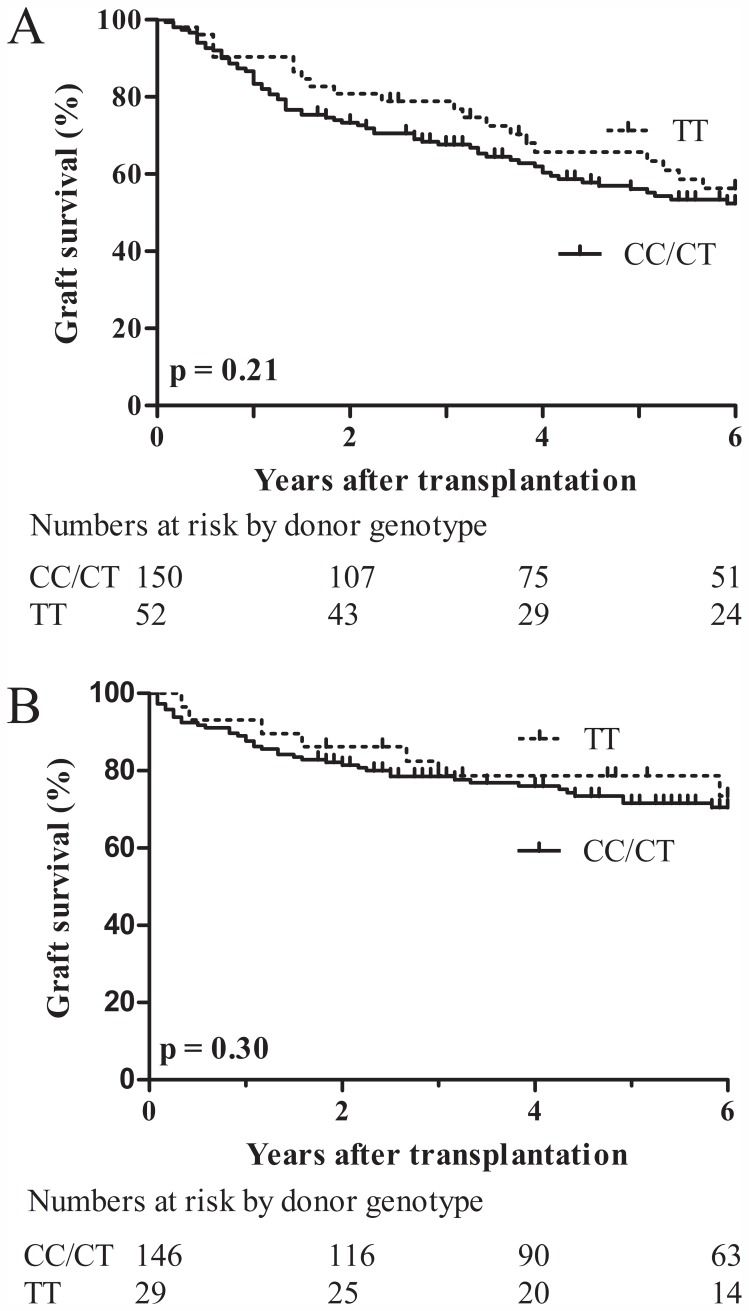
Donor IL28B genotype and graft survival by HCV status. Kaplan-Meier curves for liver graft survival in HCV infected (A) and non-HCV recipients (B).

Liver related graft loss was defined as graft loss occurring secondary to complications of portal hypertension or liver failure. 77% had jaundice/ascites; 15% fibrosing cholestatic hepatitis; 8% variceal bleeding. When survival curves were plotted using liver-related only graft loss, effect of IL28B genotype on graft survival was still evident with a graft survival of 80% in CC/CT vs. 66% in TT genotype (p = 0.006). Among non-HCV recipients, graft survival rates were 82%, 76% and 69% respectively for patients with CC, CT and TT genotypes. There was no significant difference in graft survival in non-HCV recipients with CC/CT and TT genotype (Fig. 2B). In addition, no significant differences in graft survival were observed based on donor IL28B genotype irrespective of recipient HCV status (Fig. 3A, 3B). The study did not have the power to assess the effect of matched recipient and donor TT and CC/CT genotypes on graft survival.

Multivariable Cox regression analysis was performed for the entire cohort adjusting for covariates with p<0.2 in the univariate analysis (donor and recipient age, presence of hypertension and diabetes, HCV status, HCV viral load at the time of LT, HCV genotype, HCC status, and IL28B genotype). Recipient IL28B genotype (HR for TT genotype, 1.27; 95% CI 1.04–1.56; p = 0.022), HCV status (HR 3.27; 95% CI 1.86–5.74; p<0.001), and diabetes (HR 12.86; 95% CI 5.17–32.07; p<0.001) were the only factors that significantly influenced graft survival.

We next tested the impact of SVR and IFN therapy on graft survival in the HCV population. In a univariate analysis neither SVR (HR 0.84; 95% CI 0.69–1.01; p = 0.07) nor IFN therapy (HR 0.87; 95% CI 0.73–1.03; p = 0.11) was an independent predictor for graft survival. IL28B genotype remained an independent predictor for graft survival after adjusting for SVR (HR 1.30; 95% CI 1.06–1.59; p = 0.01) or IFN therapy (HR 1.31; 95% CI 1.07–1.60; p = 0.01).

### Recipient IL28B Genotype was Associated with Time to Clinically Relevant HCV Recurrence

We tested the effect on IL28B on timing of HCV recurrence because HCV recurrence, especially within first year after LT, was associated with worse graft and patient outcomes [22]. Only patients with at least one liver biopsy were included in this analysis. Of the 327 HCV recipients, 286 (87%) had clinically warranted liver biopsies. 50% of the biopsies were performed after 3 months of LT (Fig. S1). 91% of patients that had a biopsy within 3 months of LT that did not show recurrence of HCV had at least one further biopsy 3 months after LT. 16% of HCV patients had two, 20% had three, 37% had more than three liver graft biopsies after LT.

238 (83%) patients had evidence of histological HCV recurrence. Rates of histological HCV recurrence for the entire follow-up were 81% and 92% for CC/CT and TT, respectively. There was no significant difference in the histological grading of HCV recurrence severity or fibrosis scores among the IL28B groups. Because liver biopsies were not performed at the same time point post LT time dependent variability cannot be excluded.

**Figure 4:**
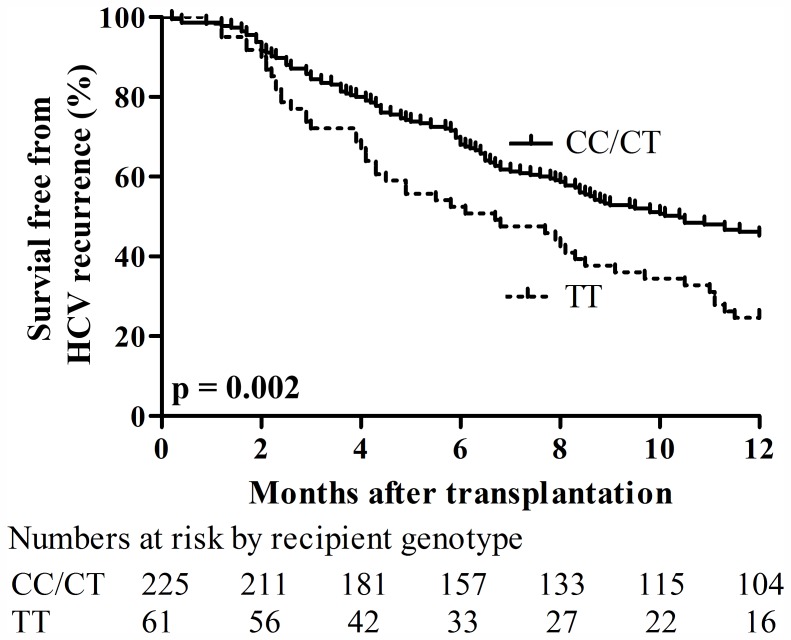
Kaplan-Meier curves for survival free from HCV recurrence according to recipient IL28B genotypes. Only patients with a liver biopsy were included in the analysis. Percentage of patients without histological HCV recurrence on clinically indicated biopsies within first year after LT was higher in CC/CT compared to TT genotype (p = 0.002).

The median time to histological HCV recurrence was significantly longer (p = 0.002) in CC/CT (10.4 months; IQR 4.8–30.6) compared to recipients with TT genotype (6.7 months; IQR 2.9–11.5). Plotted over time CC/CT recipients had significantly lower rates of histological HCV recurrence within the first year after LT compared to TT genotype (45% vs. 75%, p = 0.002; Fig. 4).

### Effect of IL28B Genotype on Sustained Virological Response (SVR)

Of 238 patients with histological HCV recurrence, 108 (45%) received treatment with pegylated interferon and ribavirin. While recipient IL28B genotype data were available for all 108 patients, donor IL28B genotype data were available for 70% of the patients. The rates of SVR were 30% in CC/CT and 17% in TT recipients (p = 0.15, Fig. S2A). The rates of SVR were 33% in CC/CT and 27% in TT donors (p = 0.5, Fig. S2B).

### IL28B Genotype had no Effect on IL28 Gene Expression

**Figure 5:**
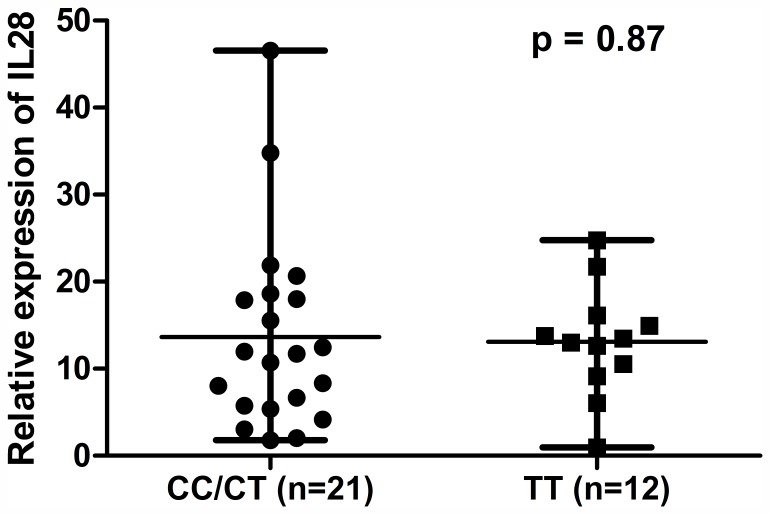
Relative expression of IL28 mRNA based on IL28B genotypes. There was no effect of IL28B genotypes on IL 28 mRNA expression. Median expression and range were shown.

We tested whether intrahepatic IL28B mRNA expression was affected by the IL28B genotypes. We observed no significant differences of the IL28 mRNA transcripts based IL28B CC/CT and TT genotypes (Fig. 5).

## Discussion

Our results showed that recipient IL28B polymorphism was linked with outcomes in patients with HCV undergoing LT. We found that while donor IL28B genotype was not associated with graft survival, the recipient IL28B TT genotype, compared to CC/CT genotypes, was associated with early and clinically relevant HCV recurrence and inferior graft survival. We also found that the detrimental effect of the TT genotype on HCV recurrence and graft survival was independent of antiviral treatment. Furthermore, we demonstrated for the first time that IL28B genotype had no effect on graft survival in a large cohort of non-HCV patients.

Our findings that effects of IL28B genotype were independent of anti-viral therapy were consistent with studies that have shown that the C allele of IL28B rs12979860, as compared to T allele, was associated with higher rates of spontaneous viral clearance [5], [23], [24]. While viral clearance is unlikely to occur after LT we hypothesize that IL28B genotype was associated with an altered innate immune response (e.g. interferon-stimulated genes) to induce an anti-viral state. This is in agreement with data that the expression of hepatic interferon-stimulated genes was strongly associated with IL28B variants producing a more favorable immunologic profile for viral clearance [25], [26]. Interestingly, in the absence of HCV infection, interferon stimulated gene expression in the liver was not associated with the IL28B genotype [27]. Confirming previous data our results showed that there was no correlation between IL28B genotypes and IL28B gene transcript levels [26]. How genetic polymorphisms affect IL28B function is not fully understood and merits further studies but our observation that IL28B genotype had no effect in the non-HCV population supports a direct or indirect anti-viral effect.

While several recent studies have reported the effects of IL28B genotype on anti-viral response after LT our study is one of the largest with a long follow up that examined the effects of IL28B on the natural history of HCV in untreated recipients [9], [11], [17], [28]. A study conducted in Europe that included 91 patients with HCV liver graft reinfection showed that the response to antiviral therapy was strongly associated with the donor’s IL28B TT genotype but only weakly with the recipient’s IL28B genotype [8]. No significant association of either recipient or donor IL28B genotype with 3 and 5-year graft or patient survival were observed [8]. Charlton et al. studied 189 HCV infected LT patients in the U.S. of whom one third were treated for recurrent HCV [9]. While the IL28B genotype of the donor and recipient were strongly and independently associated with higher rates of SVR, there was no significant difference in overall graft survival [9]. Coto-Llerena et al. tested the effects of the IL28B genotypes in 128 European donor and recipient pairs and found that the recipient genotype played a major role in the response to therapy, while donors effect depended on a favorable recipient IL28B genotype [10].

In agreement with other data we did not find an association between donor IL28B genotype and liver outcomes [17]. This is in contrast to reports suggesting an important influence of the donor IL28B genotype in HCV infected recipients [8]–[10]. In addition, others found that a favorable donor IL28B CC genotype was only noticeable after antiviral therapy [11]. As donor PBMC are often not available several studies used DNA samples obtained from implanted liver biopsies. The rapid repopulation of the graft with recipient-derived cells can confound the donor genotype determination explaining some of the discrepancies observed among different studies [30]. In contrast, our donor DNA samples were isolated from lever tissue before transplantation. While recipient IL28B genotype is an important biomarker after LT we believe that the effect of the donor IL28B on post transplant course requires further studies.

IL28B is produced by both bone marrow-derived as well as hepatocytes and the interplay between donor and recipient genotypes is complex after LT. It has been established that in murine models of LT, a proportion of the liver allografts non-parenchymal cells are recipient in origin and as such the recipient genotypes may ultimately impact long-term liver allograft function [29]. Thus, IL28B might cause an attenuation of the anti-viral state due to altered intragraft interferon-stimulated gene and IFN-k1-3 expression.

Although we found a trend towards lower rates of SVR in the IL28B TT neither recipient nor donor IL28B status were significantly associated with SVR after anti-HCV treatment as it was observed in other studies [7]–[11]. In addition, we observed 30% SVR rate in the favorable IL28B genotype which was lower compared to other studies ranging from 42 to 86%. Lack of an association and lower rate of SVR in our study might be due to ethnic differences, timing of treatment initiation, disease severity, treatment adherence, the type (deceased versus living) and quality of the donor organ (e.g. donor age). Of note our study included a higher rate of African Americans compared to other studies [8]–[10].

In our HCV cohort, the prevalence of CC genotype was similar between recipients and donors. However, prevalence of CC genotype was significantly less in HCV infected LT recipients compared to non-HCV LT recipients. This is consistent with previous findings that reported a significantly lower prevalence of CC genotype in HCV infected LT recipients [8], [9]. These findings suggest that HCV infected patients with CC genotype had better outcomes leading to enrichment of non-CC genotypes in patients that progress to end stage liver disease requiring LT.

The large sample size of consecutively enrolled patients is a major strength of our study. Furthermore, our cohort’s liver allograft survival rates at 1 and 5 years are comparable to nationwide deceased donor liver graft survival rates of 82% and 68% respectively [31]. However, given the lack of an association between IL28B genotype and graft survival in the non-HCV group, we acknowledge that our study may be underpowered to detect a possible association in this subgroup. Our study is somewhat limited by the fact that our data on HCV recurrence was based on clinically indicated biopsies rather than timed protocol biopsies. 87% of our HCV cohort underwent at least on biopsy, of these 73% had two or more biopsies reflecting our program’s low threshold for performing liver biopsies in the setting of abnormal liver chemistry tests.

In conclusion, our study demonstrated that donor IL28B genotype did not affect graft survival in contrast to recipient IL28B genotype, which was associated with graft survival, and clinically relevant HCV recurrence after LT in HCV infected recipients.

## Supporting Information

Figure S1 **Timing of first clinically indicated biopsies in the HCV cohort.** 91% of patients that had a biopsy within 3 months of LT that did not show recurrence of HCV had at least one further biopsy 3 months after LT (not shown).
(TIF)

Figure S2 **Effect of IL28B genotype on sustained virological response (SVR).** Recipient IL28B (A) and donor IL28B genotype (B) and SVR rates. There were no significant differences in the rates of SVR based on IL28B genotypes.
(TIF)
