# Supplementary figures and images for: The Association of IL28B Polymorphism and Graft Survival in Patients with Hepatitis C Undergoing Liver Transplantation
Source: PLoS One. 2013 Jan 30;8(1):e54854. doi: 10.1371/journal.pone.0054854 (PMC3559776; doi:10.1371/journal.pone.0054854)

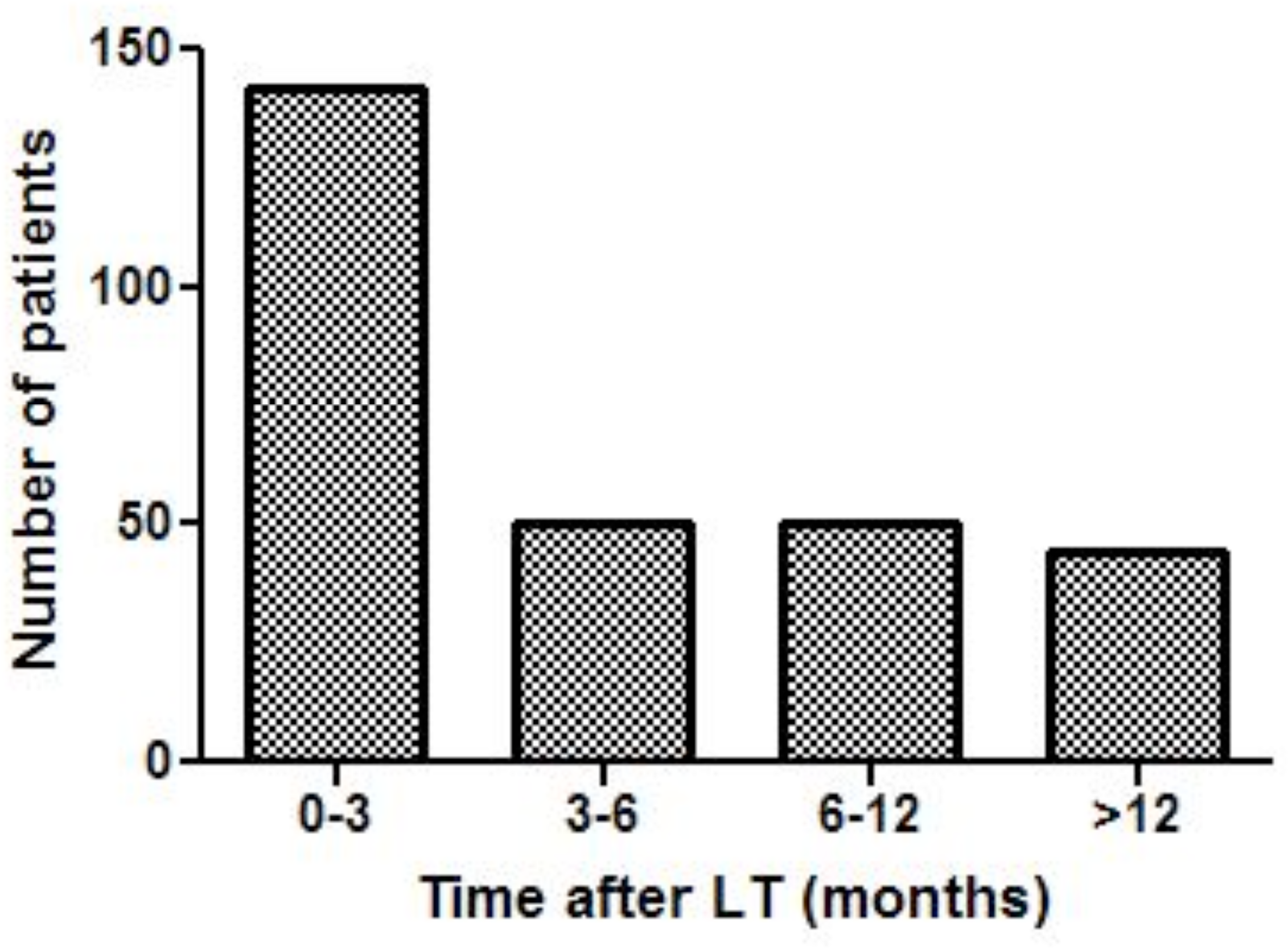

Supplement: Figure S1 — Timing of first clinically indicated biopsies in the HCV cohort. 91% of patients that had a biopsy within 3 months of LT that did not show recurrence of HCV had at least one further biopsy 3 months after LT (not shown). (TIF) [file pone.0054854.s001.tif]

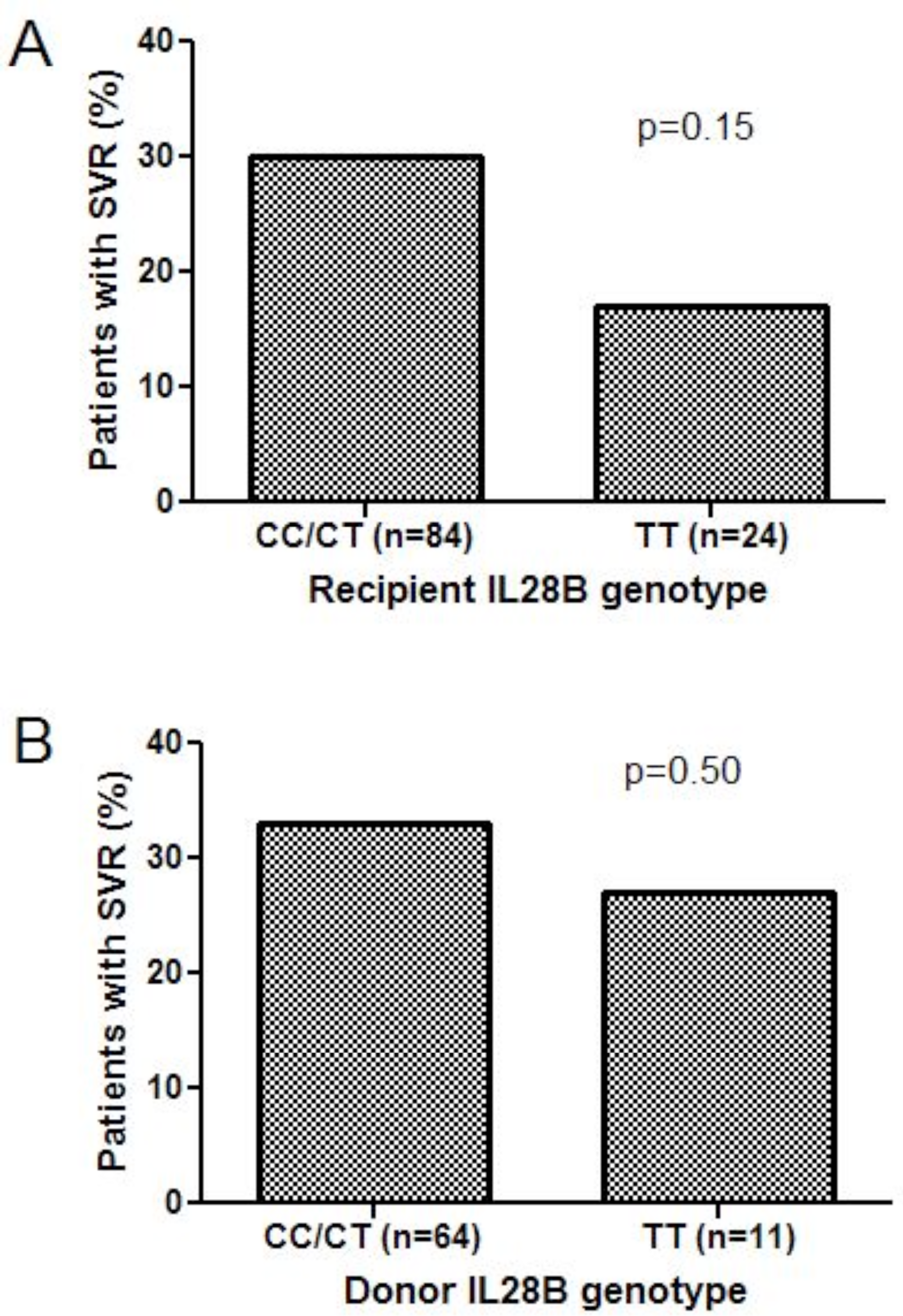

Supplement: Figure S2 — Effect of IL28B genotype on sustained virological response (SVR). Recipient IL28B (A) and donor IL28B genotype (B) and SVR rates. There were no significant differences in the rates of SVR based on IL28B genotypes. (TIF) [file pone.0054854.s002.tif]
